# Closed-loop response properties of a visual interneuron involved in fly optomotor control

**DOI:** 10.3389/fncir.2013.00050

**Published:** 2013-03-27

**Authors:** Naveed Ejaz, Holger G. Krapp, Reiko J. Tanaka

**Affiliations:** ^1^Institute of Cognitive Neuroscience, University College LondonLondon, UK; ^2^Department of Bioengineering, Imperial College LondonLondon, UK

**Keywords:** brain machine interface, optomotor control, closed-loop system, blowfly, electrophysiology, optic flow, motion vision

## Abstract

Due to methodological limitations neural function is mostly studied under open-loop conditions. Normally, however, nervous systems operate in closed-loop where sensory input is processed to generate behavioral outputs, which again change the sensory input. Here, we investigate the closed-loop responses of an identified visual interneuron, the blowfly H1-cell, that is part of a neural circuit involved in optomotor flight and gaze control. Those behaviors may be triggered by attitude changes during flight in turbulent air. The fly analyses the resulting retinal image shifts and performs compensatory body and head rotations to regain its default attitude. We developed a fly robot interface to study H1-cell responses in a 1 degree-of-freedom image stabilization task. Image shifts, induced by externally forced rotations, modulate the cell’s spike rate that controls counter rotations of a mobile robot to minimize relative motion between the robot and its visual surroundings. A feedback controller closed the loop between neural activity and the rotation of the robot. Under these conditions we found the following H1-cell response properties: (i) the peak spike rate decreases when the mean image velocity is increased, (ii) the relationship between spike rate and image velocity depends on the standard deviation of the image velocities suggesting adaptive scaling of the cell’s signaling range, and (iii) the cell’s gain decreases linearly with increasing image accelerations. Our results reveal a remarkable qualitative similarity between the response dynamics of the H1-cell under closed-loop conditions with those obtained in previous open-loop experiments. Finally, we show that the adaptive scaling of the H1-cell’s responses, while maximizing information on image velocity, decreases the cell’s sensitivity to image accelerations. Understanding such trade-offs in biological vision systems may advance the design of smart vision sensors for autonomous robots.

## INTRODUCTION

In recent years an increasing interest has emerged to apply biological principles of signal processing and control design to autonomous robotics. An enormous body of behavioral and physiological data accumulated over several decades on how the nervous system, mostly of insects, uses sensory signals for motor control (e.g., review: Taylor and Krapp, 2007) led to a significant growth in biomimetic robotics (Floreano et al., 2009; Srinivasan, 2011; Srinivasan et al., 2012). The major drive for this development comes from two directions: engineers are keen to exploit biology for the design of new robust as well as adaptive sensor and control systems, while neurobiologists are interested in robotics as a tool to validate their experimentally derived functional principles (Webb, 2008; Barth et al., 2012)

A prominent example of the joint venture between neurobiologists and engineers is the application of functional principles of insect vision to guidance, navigation, and control in aerial robotics (Srinivasan et al., 2012). Discoveries on how flies and bees process visual motion information to estimate their self-motion and control their flight has sparked a number of projects where the underlying principles were implemented in autonomous small scale air vehicles (Hyslop and Humbert, 2010; Hyslop et al., 2010). Although most control systems, both in biology and engineering operate under closed-loop conditions, many implementations so far were based on experimental data obtained under open-loop conditions.

Invertebrate animal models are ideally suited for studying the response properties of neural control circuits generating movements under both open- and closed-loop conditions. Specifically, flies display a broad repertoire of visually guided behaviors including gaze and flight stabilization reflexes which can readily be quantified at both the behavioral and the electrophysiological level. Visuo-motor stabilization behaviors or optomotor reflexes have been extensively studied at the behavioral level under both open- and closed-loop conditions (Gotz, 1964, 1968; review: Heisenberg and Wolf, 1993). Correspondingly, a great deal is now known about the open-loop response properties of a population of visual interneurons in flies, the lobula plate tangential cells (LPTCs; review: Krapp and Wicklein, 2008), which contribute to the control of optomotor reflexes (review: Hausen, 1993). However, with only a single exception (Warzecha and Egelhaaf, 1996), studies on LPTC response properties were all carried out under open-loop conditions. The specific involvement of the LPTCs in fly visual stabilization behavior naturally poses the question as to whether or not response dynamics observed under closed-loop conditions are comparable with those measured in open-loop.

Here, we compare the open- and closed-loop response properties of an identified visual interneuron, the H1-cell, which is part of a neural circuit that provides optomotor reflexes in the fly (Haag and Borst, 2001). Specifically, we compare the effect of dynamically changed image velocities and accelerations on the instantaneous spike rate of the cell under open- and closed-loop conditions. We report that the response properties are qualitatively similar under both conditions and discuss the implication of our results in the context of fly optomotor reflexes with respect to potential applications to bio-inspired control design.

## MATERIALS AND METHODS

### FLY-ROBOT INTERFACE

**FIGURE 1:**
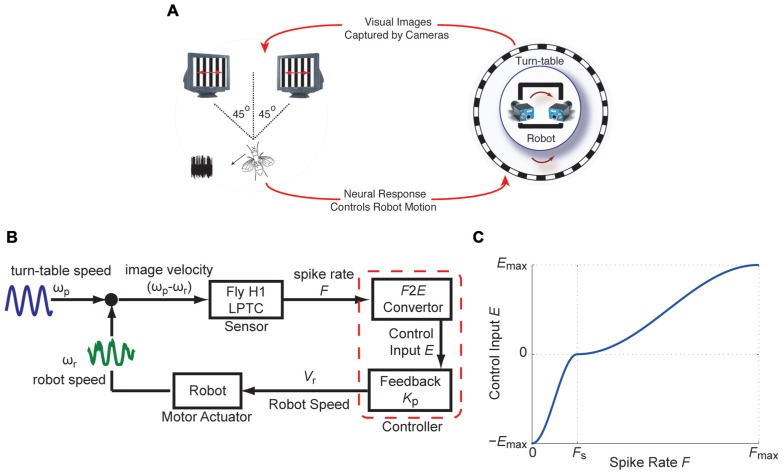
**The fly-robot interface (FRI) (A) A fly was placed in front of a visual display consisting of two high-speed CRT displays**. Input to the two monitors were provided by two high-speed video cameras mounted on a mobile robot. The robot was positioned on a turntable placed inside a cylindrical arena lined with vertically oriented grating pattern. Robot and turntable movements were limited to rotations around the vertical axis. Visual motion as a result of the rotation of the turntable was captured by the cameras. Electrophysiology recordings from the H1-cell were used to control the rotation of the robot. **(B)** Block diagram of the closed-loop FRI. Relative motion between the turn-table and the robot, ω_*p*_-ω_*r*_, caused spiking in the H1-cell. The responses of the H1-cell (instantaneous spike rate *F*), were used by a controller to compensate for externally generated turntable movements, by driving the robot in the opposite direction. **(C) **The *F2E* convertor maps *F* onto the control input *E.* The piece-wise sigmoid functions, based on which *E* was used to update the robot speed *V*_*r*_. (modified from Ejaz et al., 2012).

The closed-loop fly-robot interface (FRI; **Figure 1A**) uses the H1-cell of an immobilized fly, placed in front of two cathode ray tube (CRT) displays, as a sensor that provides an estimate of the horizontal angular velocity of a visual pattern (spatial wavelength λ_sp_ = 11°, contrast ≈ 100%). The spike rate of the H1-cell resulting from pattern motion on the CRT displays was used by closed-loop feedback controllers to regulate the angular velocity of the robot (**Figure 1B**). The robot was positioned on a turntable placed inside a cylindrical arena lined with a vertically oriented grating pattern. The dynamic properties of the robot (Arexx Engineering, ASURO Robot Kit) and the turntable represented the real-world actuator components of the FRI. Relative motion between the robot and the visual pattern forced by movement of the turntable mimicked self-motion of the animal resulting in horizontal pattern shifts. High-speed cameras mounted on the robot captured the visual image shifts at 200 fps and presented it on the visual CRT displays.

### ELECTROPHYSIOLOGY RECORDINGS

Experiments were carried out on 2–3 day old female blowflies, *Calliphora vicina*. Each animal was immobilized and its symmetrical deep pseudo-pupil (Franceschini, 1975) was used to align the head with respect to the CRT displays. Two small holes were cut in the right and left part of the animal’s rear head capsule for placement of the recording and ground electrodes, respectively. Tungsten electrodes were used to record the extracellular spike rate of the left H1-cell’s telodendritic output arborisation (Krapp et al., 2001) in the right lobula plate. An amplitude threshold was used to digitize the spike times at a resolution of 0.1 ms. The digitized spikes were convolved with a causal half-Gaussian kernel (σ_fr_ = 0.05 ms) to obtain an estimate of the instantaneous spike rate, *F*. The instantaneous spike rate, *F*, is considered to reflect the visual motion (ω_p_ - ω_r_) under closed-loop conditions and was used as an input for the two closed-loop controllers described in the section below. A video protocol of the fly preparation can be found in Ejaz et al. (2011a).

### CLOSED-LOOP CONTROLLERS

For the controller, a non-linear transformation (*F2E* converter) and a feedback gain (*K*_p_) were applied to the instantaneous spike rate, *F*, in order to obtain an 8-bit value (*V*_r_), which was used to modulate the robot’s angular velocity (ω_r_; **Figure 1B**). *F* depends on pattern motion determined by the difference between the turntable and the robot angular velocities. The *F2E* converter converts *F* into the control input *E*, based on piece-wise sigmoid functions for 0 ≤ *F* ≤ *F*_S_ and for *F*_S_ ≤ *F* ≤ *F*_max_ where *F*_s_ and *F*_max_ represent the spontaneous and maximum spike rates, respectively (**Figure 1C**). ±*E*_max_ represents the upper and lower 8-bit values, over which the robot speed is modulated. Using this controller, the robot speed is updated by:

(1) $$\begin{array}{c} V_{r}\,\left(t+1\right)=K_{p}\cdot E+V_{r}\,\left(t\right)\,. \end{array}$$

Prior to the closed-loop experiments, both *F*_s_ (mean ± SE: 19.67 ± 2.3) and *F*_max_ (mean ± SE: 78 ± 4.27) were determined in open-loop conditions for each fly, using three trails of 5 s stimulation without and with image motion in the preferred direction (PD) of the H1-cell, respectively.

As shown below in more detail, we used two different controllers to close the loop between the visual motion (ω_p_ - ω_r_) observed by the fly and the instantaneous spiking rate, *F*, of its H1-cell. The first one is a static gain controller, which consisted of a fixed feedback gain *K*_p_ and an *F2E* converter with constant *F*_max_ (Ejaz et al., 2011a,b). This controller belongs to the class of linear feedback controllers in which the control effort is proportional to the error being controlled for. In our case, the updated robot speed is proportional to the visual motion error (Eq. 1) under closed-loop conditions. Note, that an equivalent proportional controller was previously used by Warzecha and Egelhaaf (1996), to generate a feedback signal based on the differential activity of two H1-cells under closed-loop conditions. In the second controller, the condition of a fixed feedback gain was relaxed in order to obtain an adaptive gain controller. In order to achieve an adaptive feedback gain, every 50 ms, the maximum spike rate, *F*_max_ is updated over a historical time window of length Δ*T*_ws_. Continuously updating *F*_max_ scaled the sigmoid mapping between *F* and *E *during motion in the PD (*F*_s_ ≤ *F* ≤ *F*_max_; **Figure 1C**) of the H1-cell, where the updated value for the robot speed was calculated with *K*_p_ = 1 in Eq. 1. This scaling method provided the basis for the adaptive feedback gain, and was motivated by a neural coding strategy proposed by Laughlin (1994).

Once a value for the updated robot speed is estimated using either controller, it is transmitted to the robot via Bluetooth. As a result, the robot speeds up or down in order to correct for the visual motion error.

### CLOSED-LOOP EXPERIMENTS

We carried out two closed-loop experiments using the setup described above.

#### Constant input with static gain controller

In order to determine the input/output relationship for the H1-cell, we applied a constant angular velocity for 12.5 s set to

(2) $$\begin{array}{c} \begin{array}{cc} \omega_{p}= & \left\{ \begin{array}{c} 0{}^{\circ}/s\;\;for\;0\;\leq t<2.5s,\\ 144{}^{\circ}/s\;\;for\;2.5s\;\leq t\leq 15s, \end{array}\right. \end{array} \end{array}$$

and used the static gain controller to close the loop.

We measured the cell’s spike rates (*F*) and the image velocities (ω_p_ - ω_r_) for five flies with four different values of *K*_*p*_, in a total of 111 trials (12 trials for *K*_*p*_ = 0.01, 24 trials for *K*_*p*_ = 0.1, 15 trials for *K*_*p*_ = 0.5, and 60 trials for *K*_*p*_ = 1.0) and discretized them at a rate of 100 Hz.

**FIGURE 2:**
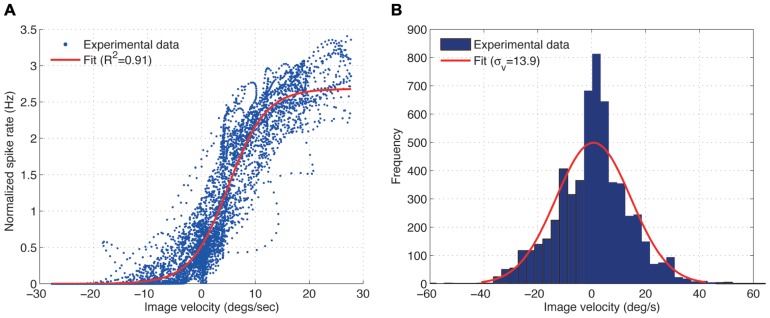
**(A)** Experimental measurement showing the input–output relationship for the H1-cell under closed-loop conditions (blue) and its least-squares sigmoid fit (*A* = 2.68, *β* = 0.29) (red), obtained from the fly robot response with the static gain controller (*K*_*p*_ = 0.1) and sinusoidal input (*f*_*i*_ = 0.03 Hz). For further explanation see text. **(B)** Distribution of the image velocities observed in closed-loop is approximately Gaussian.

**Figure 2A** shows the spike rate of the H1 cell plotted against the image velocity. A sigmoid function was fitted (least square fit) to the data shown in the plot:

(3) $$\begin{array}{c} F=\frac{A}{1+e^{-\beta \,\left(\omega_{p}-\omega_{r}\right)}}, \end{array}$$

where *A* is the upper asymptote which captures the peak spike rate and β is the growth parameter which determines the slope of the function. We use the fitting parameters *A* and β to evaluate the effect of different image velocities on the input–output relationship of the H1 cell. Larger values of *A* correspond to larger peak spike rates the cell generates for a given image velocities over the trial. The value of β specifies the slope of the function. Smaller values of β correspond to shallower and steeper slopes of the function converting image velocity into spike rate.

#### Sinusoidal input with adaptive gain controller

In order to determine the frequency response of the H1-cell, we applied sinusoidal angular velocities ω_p_ =72[sin(2Π*f_i_t*)+1] to the closed-loop system, where the input frequency *f*_i_ covered a range of 0.03 ≤ *f*_i_ ≤ 1.0 Hz, and updated the adaptive gain controller based on estimation time windows, Δ*T*_ws_ = [0.05, 0.10, 0.15] (*N* = 5 flies), to close the loop. In previous work, we showed that the adaptive gain controller has a higher cut-off frequency as compared to the static gain controller with the corresponding frequency response gains for the two controllers being approximately equal (Ejaz et al., 2012). The adaptive gain controller was therefore chosen primarily because it allowed us to obtain H1-cell responses over a wider range of frequencies as compared to the static gain controller.

At each input frequency, *f*_i_, the amplitude (power spectral densities) and phase for the H1 input (ω_p_ - ω_r_), *G*_i_ and *P*_i_, and those for the H1 output (spike rate *F*), *G*_o_ and *P*_o_, were calculated using a periodogram. Sequences were pre-multiplied with a Hamming window equal to the length of the sequence. The obtained gain $\left(\frac{G_{o}}{G_{i}}\right)$ and phase (*P*_o_ - *P*_i_) are shown in **Figures 4C,D**, respectively.

## RESULTS

We performed two experiments (described in Materials and Methods) in order to determine whether the responses of the H1-cell were different under open- and closed-loop conditions.

### EFFECTS OF THE MOMENTS OF THE IMAGE VELOCITY DISTRIBUTION ON THE H1-CELL INPUT–OUTPUT RELATIONSHIP UNDER OPEN- AND CLOSED-LOOP CONDITIONS

The input–output relationship of the H1 cell, i.e., the relationship between the image velocity (input) and the spike rate (output) was obtained for different gains, *K*_*p*_, of the static gain controller using a constant angular velocity (**Figure 2A**). Here, the spike rate was normalized by its mean over each trial. The obtained input–output relationship for the H1-cell can be approximated by a sigmoid function, as was suggested by Brenner et al. (2000) for the open-loop experiments, although the variability of the H1-cell response turned out to be much larger under our closed-loop conditions. The highly variable responses are possibly due to the non-stationarity of the image velocity distributions during closed-loop experiments. The image velocities previously used under open-loop conditions by Brenner et al. (2000) and Fairhall et al. (2001) were generated from a normal distribution with zero mean and fixed standard deviation for the duration of each trial, over which the spike rate was measured. In our closed-loop experiments, however, the image velocities observed by the fly depended on the performance of the FRI in minimizing the retinal slip speeds (ω_p_ - ω_r_). During the course of a closed-loop trial, the performance of the FRI typically varied between perfect image stabilization and short periods of high image velocities. Therefore, while the overall image velocities observed by the fly during a trial are normally distributed (**Figure 2B**), the standard deviation of the image velocities, when calculated over a shorter time interval, are constantly changing during a trial resulting in a highly variable input–output relationship of the H1-cell (**Figure 2A**).

**FIGURE 3:**
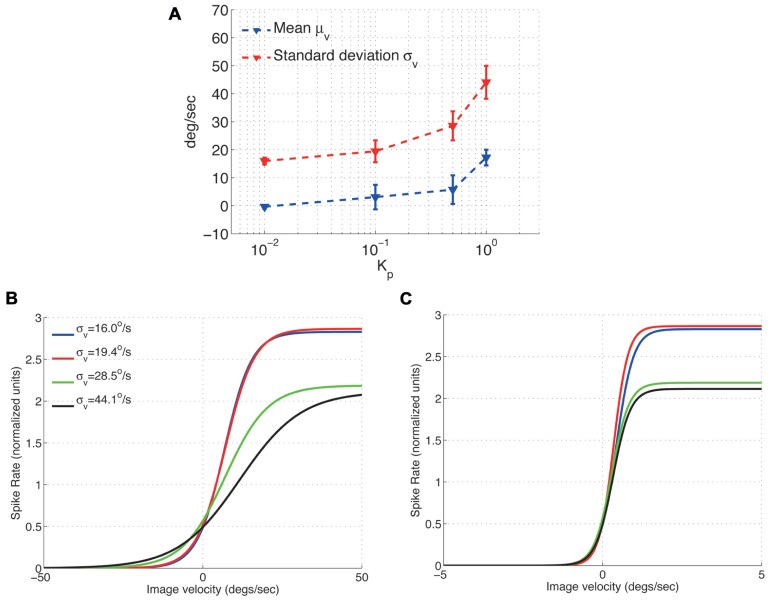
**Effect of the image velocity mean and standard deviation on the adaptive scaling properties of the H1-cell response function (A) Mean and standard deviation of the H1 input (slip speed)**. **(B)** The fitted H1-cell response functions adapts to accommodate the increased standard deviation of the image velocities **(C)** Normalizing the image velocities in **(B)** by their corresponding standard deviations results in response functions with similar slopes (around σ_v_ = 0 deg/s). The respective peak spike rates (normalized), however, remain unchanged.

To characterize the H1-cell response under closed-loop conditions, we initially measured the first (mean μ_v_) and second (standard deviation σ_v_) moments of the input, i.e., the image velocity observed by the fly (**Figure 3A**). Increasing the feedback gain *K*_*p*_ monotonically increases both μ_v_ and σ_v_. The increase in σ_v_ can be explained by control oscillations particularly pronounced for high feedback gains (Ejaz et al., 2011a). Such control oscillations are not specific to the FRI, but have also been observed as yaw torque fluctuations during closed-loop optomotor tasks in *Drosophila *(Mayer, 1989; Wolf and Heisenberg, 1990; Warzecha and Egelhaaf, 1996). The increase in μ_v_ can be explained by the fact that we use a single H1-cell for closed-loop control. Ideally, a fly would attempt to maintain optomotor equilibrium by balancing clockwise and counter-clockwise rotations so that the observed image motion is minimized. The two H1-cells would contribute sensitivity to motion in opposing directions based on which the optomotor equilibrium is maintained. However, when a single H1-cell is used for closed-loop control, the optomotor equilibrium is un-balanced which in turn leads to an increased value of μ_v_. An un-balanced optomotor equilibrium does not, however, seem to have drastic behavioral consequences for the fly. In a behavioral study, Kern and Egelhaaf (2000) occluded one eye in *Lucilia* and measured the turning responses in both freely flying and walking flies inside a visual arena. The authors concluded that it was hard to tell from the turning responses that the fly had been limited to the use of monocular vision and that while the flies exhibited a slight turning preference toward the stimulated eye (i.e., increased μ_v_), no such asymmetry could be observed in individual responses. As a result, while increasing μ_v_ decreases the overall performance of image stabilization under closed-loop, it does not affect the conclusions that can be drawn regarding the response properties of the H1-cell.

After characterizing the input to the H1-cell by its standard deviation and mean, we investigated the effect of σ_v_ on the cell’s response properties. Its input–output relationship has previously been shown in open-loop measurements to adapt to the standard deviation, σ_v_, of the input image velocity distribution (Brenner et al., 2000; Fairhall et al., 2001). Large values of σ_v_ cause the input–output function to expand along the *x*-axis (image velocity) leading to a shallower slope, i.e., smaller value for β, for the response function. In comparison, small values of σ_v_ cause compression along the velocity axis resulting in a steeper slope for the response function and consequently a higher value for β. Our experiments show that the response function also scales in proportion to the standard deviation of the image velocity under closed-loop conditions (**Figures 3B,C**). As reported by Brenner et al. (2000) for open-loop condition, normalizing the input–output relationship by the standard deviation removes differences in cell’s adaptation properties under closed-loop condition as well (**Figure 3C**).

Note however, that this normalization does not change the peak spike rate, *A*, in our closed-loop experiments (**Figure 3C**), suggesting that the peak spike rate of the H1-cell may be controlled by another moment of the image velocity distribution, possibly its mean. Further open-loop experiments on the H1-cell have shown that increasing either the mean (Reisenman et al., 2003) or the standard deviation (Borst et al., 2005) of the image velocity results in a decrease of its peak spike rate. In our closed-loop experiments, an approximate 2-fold increase in the standard deviation (from σ_v_ = 16.0 deg/s to σ_v_ = 28.5 deg/s) results in a spike rate deduction of approximately 18% (**Figure 3C**). Such a decrease is larger than it would be predicted for an increased standard deviation under open-loop conditions (Borst et al., 2005). This suggests that the peak spike rate of the H1-cell under closed-loop conditions depends on both the mean and the standard deviation of the image velocity.

The effects of the moments of the input distribution on the spike rate of the H1-cell for the open- and closed-loop conditions are highly similar in that (i) the H1-cell response is adjusted to the standard deviation of the image velocity and (ii) the H1-cell decreases its peak spike rate when mean and standard deviation of the image velocity distribution are increased.

### EFFECTS OF INCREASING IMAGE ACCELERATIONS ON THE GAIN OF THE H1-CELL RESPONSE UNDER OPEN- AND CLOSED-LOOP CONDITIONS

**FIGURE 4:**
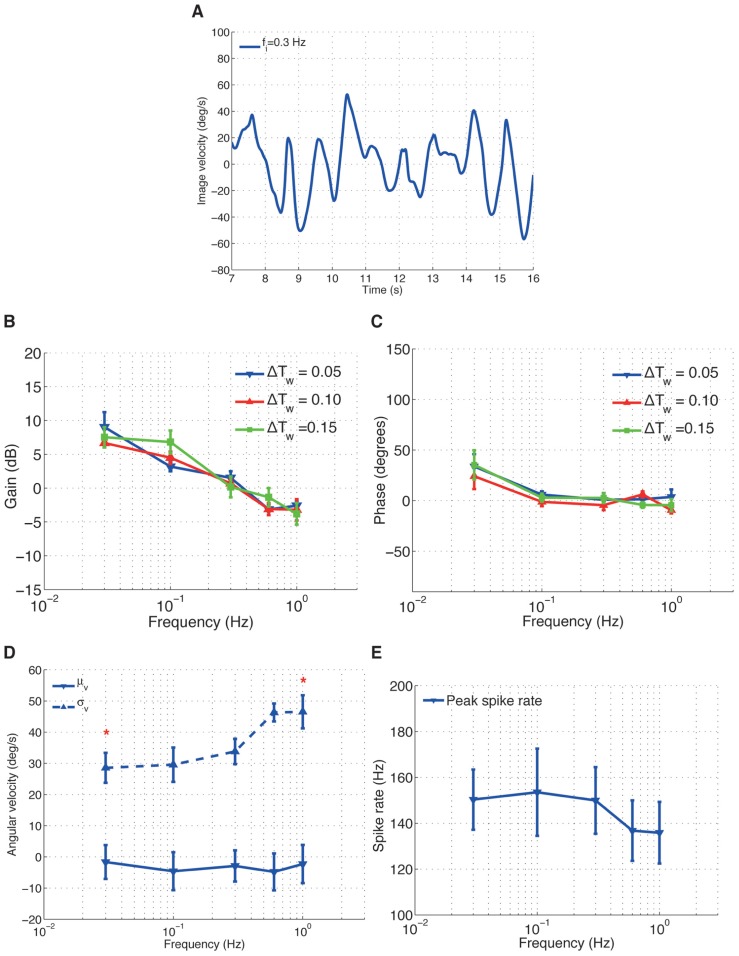
**H1-cell frequency response**. **(A)** An example time course data of image velocity observed by the fly for an input frequency *f*_i_ = 0.3 Hz. The gain **(B)** and phase **(C)** plots over different input frequencies. **(D)** The mean (μ_v_) and standard deviation (σ_v_) of the image velocities. *Values of σ_v_ for *f*_i_ = 0.3 Hz and *f*_i_ = 1.0 Hz are significantly different (calculated using Wilcoxon rank-sum method with α = 0.001). **(E)** Peak spike rate (*) for different input frequencies.

In the second experiments, we induced sinusoidal angular velocity perturbations into the closed-loop system, while varying the length of the spike rate estimation time window Δ*T*_ws_ for the adaptive gain controller. For each trial, the resulting closed-loop image velocities (**Figure 4A**) and H1-cell spike rates were recorded to investigate the cell’s frequency response.

The gain of the H1-cell decreased linearly with increasing input frequencies, with a gradient of approximately 8–9 dB/dec (**Figure 4B**). The linear decrease in gain with frequency did not, by and large, depend on the time window Δ*T*_ws_ for the adaptive controller. The corresponding phase (**Figure 4C**) of the H1-cell decreased from approximately 35° at a frequency of *f*_*i*_ = 0.03 Hz to around 0° for *f*_*i*_ > 0.03 Hz.

We are tempted to argue here that, under closed-loop conditions, the frequency-dependent decrease of the H1-cell response gain (**Figure 4C**) is related to the increase in image acceleration. For the sinusoidal image velocity perturbations we used in the second experiment, the increase in the input frequency, *f*_*i*_, leads to an increase in the image acceleration. Therefore, the gain plot of the H1-cell (**Figure 4B**) represents the relationship between the cell’s spike rate and the image accelerations under closed-loop conditions and suggests that the response gain of the cell decreases for increasing image acceleration. Such an approximately linear decrease in response gain of the cell with increasing accelerations was also observed under open-loop conditions (Borst et al., 2005).

The effects of increasing the frequency on the moments of the image velocity distribution are shown in **Figures 4D,E**. As the frequency increased, σ_v_ increased from around 28° to 46°/s (**Figure 4D**) and the peak spike rate decreased (**Figure 4E**). Actually, the decrease in the peak spike rate is largely the result of the increase in σ_v_, as the corresponding mean (μ_v_) of the image velocities is very close to 0°/s for the frequency range we examined (**Figure 4D**). It should be noted that increasing σ_v_ is equivalent to increasing the image velocity amplitudes and therefore produces higher image accelerations, which in turn decreases the response gain of the cell.

## DISCUSSION

### THE FRI AS A CLOSED-LOOP EXPERIMENTAL SYSTEM

The use of a robotic controller to understand animal behavior provides real-world physical interactions typically missing from modeling studies where a low-pass filter is used to describe the dynamics of the fly flight motor system. As argued by Webb (2006), this lack of physical interaction would mean that complex motion dynamics such as slipping due to friction cannot be accounted for in the computer model. Indeed, recent work by Dickson et al. (2010) showed that both body-inertia and -damping play a significant role in the dynamics of saccadic yaw turns in *Drosophila* flight. While the configuration of the fly in such a closed-loop experimental setup is far removed from conditions during natural flight, the stimulus velocity distributions observed by the fly in the FRI are within range of those used in previous measurements of the H1 cell under open-loop conditions (Warzecha and Egelhaaf, 1996; Brenner et al., 2000; Borst, 2003; Borst et al., 2005).

While H1-cell responses have been studied extensively under open-loop conditions (e.g., Maddess and Laughlin, 1985; Brenner et al., 2000; Borst, 2003; Reisenman et al., 2003), this paper presents the first study of the cell’s response properties for a variety of image velocity profiles under closed-loop conditions. Our FRI was used to generate dynamic visual stimuli, i.e., sinusoidal and constant image velocity perturbations to drive the responses of the H1-cell.

In a pioneering study, Warzecha and Egelhaaf (1996) obtained electrophysiology recordings from both the ipsi- and the contra-lateral H1 LPTC’s while the fly compensated for externally imposed visual motion under closed-loop conditions. In that study, however, comparatively small and constant image velocities (18) were used and thus there was little or no modulation of the image accelerations presented to the H1-cells. As a result Warzecha and Egelhaaf (1996) characterized the cell’s closed-loop responses only for a rather narrow velocity profile, compared to the dynamic visual stimuli generated by our FRI. While they found the responses of the cell to decrease as the image velocity increased which is in agreement with our findings – they did not observe the dependence of the H1-cell responses on image accelerations we report here.

A limitation of our experiments was that only the activity of one H1-cell had been considered for closed-loop control. During walking and free-flight, a fly receives information about its yaw rotation from both the ipsi- and the contra-lateral H1-cells. Using only the activity of a single cell for visual stabilization reduced the fly’s sensitivity to yaw rotations. Given that the peak spike rate of the H1-cell has been found to decrease strongly with an increase of the mean image velocity, in both open- (Reisenman et al., 2003) and closed-loop (**Figure 3B**) measurements, one key function of two H1-cells could be to keep the fly in optomotor equilibrium by trying to minimize the mean image velocity. Such a strategy of minimizing the mean image velocity would remove any restrictions on the peak spike rate of the cell. This in turn would be advantageous as the fly would remain sensitive to differences in image velocities as opposed to absolute values, which appears to be a general feature of biological sensing (Taylor and Krapp, 2007).

Our results with the FRI show that the open- and closed-loop responses are qualitatively similar, in the sense that the H1-cell maximizes the information transmitted about the image velocity distribution by adapting its input–output relationship (Brenner et al., 2000; **Figures 3B,C**). We found in addition that higher image acceleration, as a result of increased standard deviation of the image velocity distribution, decreases the gain of the H1-cell. It is important to note that this dependence is not the result of the dynamic properties of the robot or the turntable. This is because the decrease in the H1-cell response gain is too large (8–9 dB), even for small changes in acceleration (between 0.03 and 0.3 Hz), to be explained by the frequency response of either the robot or the turntable (Ejaz et al., 2011b, 2012).

In the following we will discuss our findings in more detail with an emphasis on coding of visual motion information optomotor control and the translation of closed-loop results into biomimetic applications.

### THE DECREASE IN THE RESPONSE GAIN FOR INCREASING IMAGE ACCELERATION IS A DIRECT CONSEQUENCE OF THE ADAPTIVE SCALING PROPERTY OF H1

A key finding of our closed-loop experiments we report here, is that the decrease in sensitivity to image accelerations is a direct result of the H1-cell’s adaptive scaling property. To the extent of our knowledge, this relationship has not been explicitly highlighted previously in open-loop measurements and is discussed below.

**FIGURE 5:**
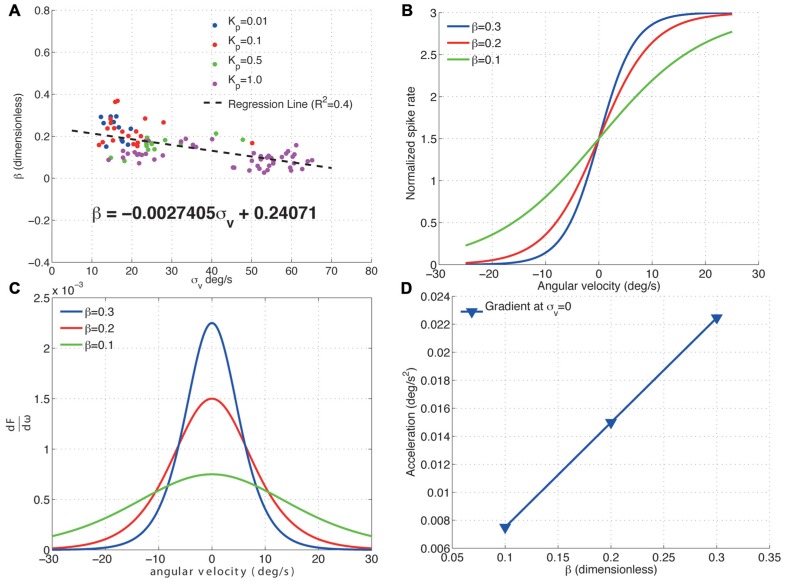
**Effect of σ_v_ on the acceleration sensitivity of the H1-cell**. **(A)** The fitted values of β for the proportional controller with gain *K*_p_ are plotted against the standard deviation of the velocity distribution, σ_v_. Increasing σ_v_ linearly decreases β as per the relationship specified by the regression line. **(B)** The input–output functions for three different values of β (normalized by the peak spike rate, *F*) show that **(C)** decreasing beta linearly decreases the gradient at the point σ_v_ = 0 deg/s **(D)** and this decrease is linear. This reduction in gradient of the H1-cell input–output function represents a decrease in sensitivity to changes in the image velocity i.e., a decrease in sensitivity to image acceleration. The results show that increasing σ_v_ directly decreases the sensitivity of the H1-cell to image accelerations.

**Figure 5** shows how the H1 cell decreases its sensitivity to acceleration by scaling its response range to fit that of a wider image velocity distribution. Increasing the standard deviation of the image velocities results in a decrease in the gradient, β, of the cells input–output function (**Figures 5A,B**). This decrease in β directly results in a linear decrease in the peak acceleration sensitivity of the cell (**Figures 5C,D**). Furthermore, this decrease in sensitivity to acceleration is linear. The adaptive re-scaling of the H1-cell responses which maximizes information transfer, apparently comes at the expense of a reduced sensitivity to image acceleration. It is tempting to speculate that the trade-off between maximizing information transmission related to the input image velocity and the reduced sensitivity to acceleration might reflect a more general strategy preferred during the evolution of sensory systems. While in the visual system a decreased sensitivity to acceleration might be partly compensated for by signals from other sensory modalities (e.g., the halteres), a decrease in information transmission would be detrimental for the neural representation of visual motion. Given that neurons are required to process information under very strict energy constraints (Laughlin et al., 1998; Laughlin, 2001), inefficiencies in neural coding might come at a high evolutionary cost. In addition, inefficient coding at the sensory system level will most certainly propagate downstream to produce inadequate motor outputs. Altogether, a loss of acceleration sensitivity as a result of adaptive re-scaling might be a comparatively small cost to pay.

The dependence of the H1-cell frequency response on image acceleration is also found to be qualitatively similar for open- and closed-loop studies. In earlier work, Brenner et al. (2000) showed that the altering the image velocity or acceleration resulted in a modulation of the responses of the H1 cell under open-loop conditions. In subsequent studies open-loop studies, the gain of the H1 cell was proposed to depend on acceleration and other higher-order time derivatives of image velocity (Borst, 2003; Borst et al., 2005). Specifically, the responses of the H1 cell decreased as a result of increasing image accelerations, which is also what we report under closed-loop conditions (**Figure 5**).

**FIGURE 6:**
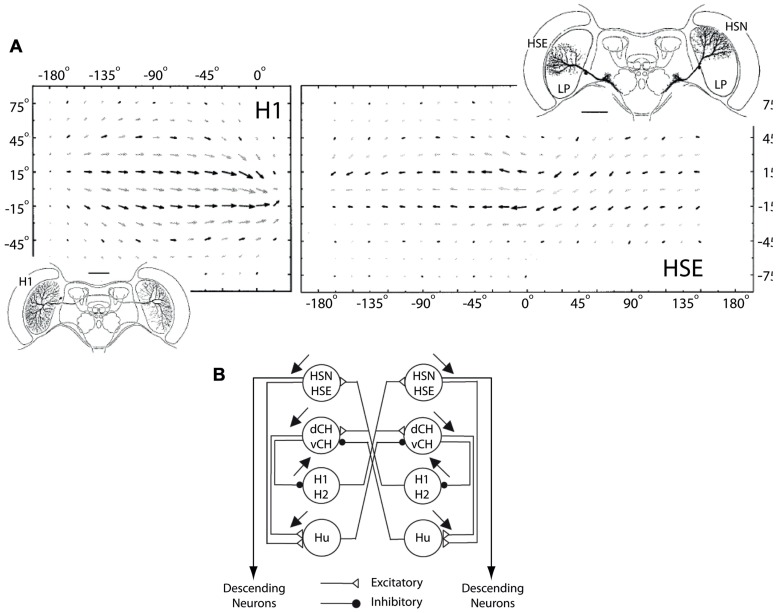
**H1 and HSE receptive fields and horizontal network connections.** **(A)** Top row shows monocular and binocular receptive fields of the H1 and the HSE LPTCs, respectively. The insets show the dendritic arborization patterns of both cells in the left lobula plate as well as the HSN LPTC in the right lobula plate. The dendritic input arbourizations and the telo-dendritic output arborizations of the H1 cell are connected via a thin axon that transmits visual motion information from the left to the right lobula plate using action potentials. The HSE and the HSN cells arborize in the equatorial and the north sections of the lobula plate, respectively (modified from Krapp et al., 2001). **(B)** The connectivity in the network of LPTCs sensitive to horizontal motion. Excitatory and inhibitory interactions are depicted with open triangles and filled circles, respectively. The HSE and HSN cells receive excitatory input from the contralateral H1 and H2 cells and project onto descending neurons which in turn supply the neck and flight motor systems of the fly (modified from Krapp et al., 2001).

Our results also show that the acceleration sensitivity of the H1-cell is highest while there is little or no pattern motion (σ_v_ = 0^o^/s, **Figure 6C**). This is clearly advantageous for the fly, as it enables the H1-cell to respond more quickly to image motion that rapidly changes from null direction to PD, as Lewen et al. (2001) proposed earlier.

While maintaining a high sensitivity to acceleration, i.e., high value of β, might make sense intuitively, it comes with potential risks. An input–output response function with a high value of β means that very small changes in angular velocity result in large changes of spike rate. β therefore partly determines the forward gain in the motion vision pathway of the system. The potential risk, however, is that with a high forward gain in combination with inherent noise in the system may easily drive the responses of downstream neural circuits into saturation. Additionally, if the feedback gain (on top of the forward gain of the H1 cell) is too high and control delays are too long, then the feedback control system is in danger of becoming instable. In this context, decreasing sensitivity to acceleration by having a lower response gain for increasing frequencies is possibly advantageous from a control theoretic point of view and this argument is discussed in the following section.

### SIMILARITY IN H1-CELL RESPONSES UNDER OPEN- AND CLOSED-LOOP CONDITIONS AND ITS IMPLICATIONS FOR OPTOMOTOR CONTROL

As a hetero-lateral neuron, the H1 cell helps disambiguate between rotation- and translation-induced optic flow as it is completely inhibited during forward translation but excited during yaw rotations. The cell also provides excitatory input to the HSE and HSN cells (**Figure 6**), two major output neurons of the visual system that respond to visual motion with a graded modulation of their membrane potential (Hausen, 1976, 1982). By connecting to the contralateral HSE and HSN cells, it makes the response of these output cells more specific to yaw rotation. Therefore, the response properties and connectivity of the H1 cell make it an important neuron in the optomotor pathway of the fly.

It is by no means trivial that the response of the H1-cell to the moments of the image velocity distribution (mean, standard deviation, acceleration), are highly similar under open- and closed-loop conditions. This similarity may reflect the way in which the sensory-motor control loops are organized in the fly.

One particular model of the sensory-motor control loop in the fly proposed by Warzecha and Egelhaaf (1996) and Borst et al. (2005) does not require sensory feedback to explain the non-linear response properties of the H1-cell. In this model, the non-linear properties of the H1-cell and the LPTCs in general, can be predicted solely based on the properties of the Reichardt (1987) elementary movement detector (EMD). Warzecha and Egelhaaf (1996) suggested that the reduced gain of the H1-cell at higher image velocities is the result of intrinsic response properties of EMDs. Similarly, Borst et al. (2005) showed that an EMD model could explain the dependence of the H1-cell responses to the standard deviation and the autocorrelation time constant of the image velocities. In both cases, no feedback signals were required to explain the non-linear response properties of the H1-cell, which were suggested to be based on the computational structure of the EMD model. This particular model of control architecture is closely linked to that proposed by Wehner (1987) who argued that architecture and response properties of invertebrate sensory systems reflect a detailed model of the physical world. If the model is true, no feedback signals are necessary and H1-cell responses under closed-loop are simply the result of EMD properties, readily observable under open-loop conditions.

An alternate control architecture involves forward models, in which a copy of a motor command (efference copy) is used to subtract those components of the sensory feedback that are due to the animal’s own action (Chan et al., 1998; Wolpert and Ghahramani, 2000; reviews: Webb, 2004; Krapp and Wicklein, 2008). Forward models or efference copies have been proposed to explain the mechanism by which flies adjust their gain parameters when faced with unexpected visual feedback during an optomotor task inside a flight simulator (Kirschfeld, 1989). One possible explanation for the similarity of the H1-cell responses under open and closed-loop conditions could be that in fully restrained animals an efference copy from the motor system may not be sent to the LPTCs. Our FRI did not allow us to assess the potential impact of an efference copy signal. Even though the fly was under closed-loop conditions, it was immobilized for the purpose of obtaining electrophysiology recordings which makes it unlikely that the animal would have generated motor commands similar to those under free flight conditions. At this point, we can only speculate on which control architecture, i.e., with or without feedback control at the level of the LPTCs, best explains the similarity in H1-cell responses as measured under open- and closed-loop conditions.

Finally, in the context of optomotor control, the frequency response of the H1-cell (**Figures 4C** and **5D**) imposes certain limitations on fly’s ability to compensate for externally imposed yaw rotations. For the visual system to contribute to the stabilization of visual motion, the reduction of gain and cut-off frequency of the horizontal cells in the lobula plate must be higher than those of the flight muscles which produce compensatory torque. The response delay in the motion vision pathway (≈30 ms) for the fly is long compared to other sensory systems like the ocelli (≈15 ms) and the halteres (≈10 ms) (Taylor and Krapp, 2007). It would therefore make sense for the cell’s response not to have a high gain at high frequencies that would potentially result in instabilities of the control system as initially proposed by Warzecha and Egelhaaf (1996). A low gain at high frequencies would be in agreement with the proposed primary function of LPTCs to mainly compensate for slow drifts (Collett et al., 1993). In comparison, the halteres and the ocelli, with their short response delays, would be better suited to control yaw rotations in the higher dynamic range. The importance of keeping delays to a minimum within the optomotor control loop, and in biological control loops, in general (Dickson et al., 2010), is also evidenced by our finding that the response phase of the H1-cell stays close to zero over the tested frequency range.

The surprising qualitative similarity between closed- and open-loop data suggest that it is reasonable, in instance first approximation, to base any implementations of fly inspired (optomotor) control design on experimental open-loop data. This could potentially expedite the translation of biological design principles in technical applications as methodologically more challenging closed-loop experiments may not always be required to conclusively characterize the dynamics of neuronal responses. It should be noted, however, that the present study focused only on a 1 DoF visual stabilization task. Neuronal closed- and open-loop activity supporting multisensory control of higher dimensional tasks may as well show very different response dynamics – in particular if observed in freely or semi-freely moving animals.

## Conflict of Interest Statement

The authors declare that the research was conducted in the absence of any commercial or financial relationships that could be construed as a potential conflict of interest.

## References

[B1] Barth F. Humphrey J. A. Srinivasan M. V. (2012). *Frontiers in Sensing: From Biology to Engineering*. Heidelberg: Springer

[B2] Borst A. (2003). Noise, not stimulus entropy, determines neural information rate. *J. Comput. Neurosci.* 14 23–31 12435922 10.1023/a:1021172200868

[B3] Borst A. Flanagin V. L. Sompolinsky H. (2005). Adaptation without parameter change: dynamic gain control in motion detection. *Proc. Natl. Acad. Sci. U.S.A.* 102 6172–6176 15833815 10.1073/pnas.0500491102 PMC1087925

[B4] Brenner N. Bialek W van Steveninck R. D. R. (2000). Adaptation rescaling maximizes information transmission. *Neuron* 26 695–702 10896164 10.1016/s0896-6273(00)81205-2

[B5] Chan W. P. Prete F. Dickinson M. H. (1998). Visual input to the efferent control system of a fly’s “gyroscope”. *Science* 280 289–292 9535659 10.1126/science.280.5361.289

[B6] Collett T. Nalbach H. Wagner H. (1993). “Visual stabilization in arthropods,” in *Visual Motion and its Role in the Stabilization of Gaze,* eds Miles F. A. Wallman J. (Amsterdam: Elsevier) 239–263 8420551

[B7] Dickson W. B. Polidoro P. Tanner M. M. Dickinson M. H. (2010). A linear systems analysis of the yaw dynamics of a dynamically scaled insect model. *J. Exp. Biol.* 213 3047–3061 20709933 10.1242/jeb.042978

[B8] Ejaz N. Peterson K. D. Krapp H. G. (2011a). An experimental platform to study the closed-loop performance of brain-machine interfaces. *J. Vis. Exp.* 10 3791 10.3791/1677 PMC3143588 21445031

[B9] Ejaz N. Tanaka R. J. Krapp H. G. (2011b). “Closed-loop performance of a proportional controller for visual stabilization using a fly-robot interface,” in *IEEE International Conference on Robotics and Biomimetics (ROBIO)* December 7–11, 2011, Karon Beach 1509–1515

[B10] Ejaz N. Tanaka R. J. Krapp H. G. (2012). “Static versus adaptive gain control strategy for visuo-motor stabilization,” in *Living Machine 2012: The International Conference on Biomimetic and Biohybrid Systems* Vol. 7375 eds Prescott T. J. Lepora N. F. Mura A. Verschure P. F. M. J. (Berlin: Springer) 107–119

[B11] Fairhall A. L. Lewen G. D. Bialek W. Ruyter van Steveninck R. R. D. (2001). Efficiency and ambiguity in an adaptive neural code. *Nature* 412 787–792 11518957 10.1038/35090500

[B12] Floreano D. Zufferey J. C. Srinivasan M. V. Ellington C. (eds). (2009). *Flying Insects and Robots*. Berlin: Springer-Verlag

[B13] Franceschini N. (1975). “Sampling of the visual environment by the compound eye of the fly: fundamentals and applications,” in *Photoreceptor Optics* eds Snyder A. W. Menzel R. (Berlin: Springer) 98–125

[B14] Gotz K. G. (1964). Optomotorische untersuchung des visuellen systems einiger augenmutanten der fruchtfliege *Drosophila*. *Kybernetik* 77–92 5833196 10.1007/BF00288561

[B15] Gotz K. G. (1968). Flight control in *Drosophila* by visual perception of motion. *Kybernetik* 4 199–208 5731498 10.1007/BF00272517

[B16] Haag J. Borst A. (2001). Recurrent network interactions underlying flow-field selectivity of visual interneurons. *J. Neurosci.* 21 5685–5692 11466440 10.1523/JNEUROSCI.21-15-05685.2001 PMC6762678

[B17] Hausen K. (1976). Functional characterisation and anatomical identification of motion sensitive neurones in the lobula plate of the blowfly *Calliphora erythrocephala*. *Z Naturforsch* 31 629–633

[B18] Hausen K. (1982). Motion sensitive interneurons in the optomotor system of the fly. II. The horizontal cells: receptive field organization and response characteristics. *Biol. Cybern.* 46 67–79

[B19] Hausen K. (1993). “Decoding of retinal image flow in insects,” in *Reviews of Oculomotor Research: Visual motion and its role in the stabilisation of gaze* eds Miles F. A. Wallman J. (Amsterdam: Elsevier) 203–235 8420550

[B20] Heisenberg M. Wolf R. (1993). “The sensory-motor link in motion dependent flight control of flies” in *Visual Motion and its Role in the Stabilization of Gaze* eds Miles F. A. Wallman J. (Amsterdam: Elsevier) 265–283 8420552

[B21] Hyslop A. Krapp H. G. Humbert J. S. (2010). A control theoretic interpretation of directional motion preferences in optic flow processing interneurons. *Biol. Cybern.* 103 353–364 20694561 10.1007/s00422-010-0404-8

[B22] Hyslop A. M. Humbert J. S. (2010). Autonomous navigation in 3-D urban environments using wide field integration of optic flow. *J. Guid. Control Dyn.* 33 147–159

[B23] Kern R. Egelhaaf M. (2000). Optomotor course control in flies with largely asymmetric visual input. *J. Comp. Physiol.* 186 45–55 10659042 10.1007/s003590050006

[B24] Kirschfeld K. (1989). Automatic gain control in movement detection of the fly: implica- tions for optomotor responses. *Naturwis* 76 378–380

[B25] Krapp H. G. Hengstenberg R. Egelhaaf M. (2001). Binocular contributions to optic flow processing in the fly visual system. *J. Neurophysiol.* 85 724–734 11160507 10.1152/jn.2001.85.2.724

[B26] Krapp H. G. Wicklein M. (2008). “Central processing of visual information in insects,” in *The Senses: A Comprehensive Reference* eds Masland R. Albright T. D. (San Diego: Academic Press) 131–204

[B27] Laughlin S. B. (1994). Matching coding, circuits, cells, and molecules to signals – general principles of retinal design in the fly’s eye. *Prog. Retin. Eye Res.* 13 165–196

[B28] Laughlin S. B. (2001). Energy as a constraint on the coding and processing of sensory information. *Curr. Opin. Neurobiol.* 11 475–480 11502395 10.1016/s0959-4388(00)00237-3

[B29] Laughlin S. B. van Steveninck R. D. R. Anderson J. C. (1998). The metabolic cost of neural information. *Nat. Neurosci.* 1 36–41 10195106 10.1038/236

[B30] Lewen G. D. Bialek W Ruyter van Stevenick R. R. D. (2001). Neural coding of naturalistic motion stimuli. *Network* 12 317–329 11563532

[B31] Maddess T. Laughlin S. B. (1985). Adaptation of the motion-sensitive neuron H1 is generated locally and governed by contrast frequency. *Proc. R. Soc. Lond. B Biol. Sci.* 225 251–275

[B32] Mayer M. (1989). *Raumliche und zeitliche integration von bewegungsinformatiiomn visuellenf lugsystemv on Drosophila melanogaster. modellentwicklunagu f der grundlagev on verhaltens-versuchen*. Doctoral dissertation, Universität Würzburg, Würzburg

[B33] Reichardt W. (1987). Evaluation of optical motion information by movement detectors. *J. Comp. Physiol.* 161 533–547 3681769 10.1007/BF00603660

[B34] Reisenman C. Haag J. Borst A. (2003). Adaptation of response transients in fly motion vision I: Experiments. *Vis. Res.* 43 1291–1307 12726835 10.1016/s0042-6989(03)00091-9

[B35] Srinivasan M. V. (2011). Honeybees as a model for the study of visually guided flight, navigation, and biologically inspired robotics. *Physiol. Rev.* 91 413–460 21527730 10.1152/physrev.00005.2010

[B36] Srinivasan M. V. Moore R. J. D. Thurrowgood S. Soccol D. Bland D. (2012). “From biology to engineering: insect vision and applications to robotics,” in *Frontiers in Sensing: From Biology to Engineering* eds Barth F. G. Humphrey J. A. C. Srinivasan M. V. (Berlin: Springer-Verlag) 19–39

[B37] Taylor G. K. Krapp H. G. (2007). Sensory systems and flight stability: what do insects measure and why? *Adv. Insect Physiol.* 34 231–316

[B38] Warzecha A. K. Egelhaaf M. (1996). Intrinsic properties of biological motion detectors prevent the optomotor control system from getting unstable. *Philos. Trans. R. Soc. Lond. B Biol. Sci.* 351 1579–1591

[B39] Webb B. (2004). Neural mechanisms for prediction: do insects have forward models. *Trends Neurosci.* 5 278–282 15111010 10.1016/j.tins.2004.03.004

[B40] Webb B. (2006). Validating biorobotic models. *J. Neural Eng.* 3 25–35 10.1088/1741-2560/3/3/R01 16921200

[B41] Webb B. (2008). Automatic gain control in movement detection. *Adv. Study Behav.* 38 1–58

[B42] Wehner R. (1987). Matched filters: neural models of the external world. *J. Comp. Phys. A* 161 511–531

[B43] Wolf R. Heisenberg M. (1990). Visual control of straight flight in Drosophila melanogaster. J. Comp. Physiol. A 167 269–283 2120434 10.1007/BF00188119

[B44] Wolpert D. M. Ghahramani Z. (2000). Computational principles of movement neuroscience. *Nat. Neurosci*. 3 1212–1217 11127840 10.1038/81497

